# The rise of intelligent research: how should artificial intelligence be assisting researchers in conducting medical literature searches?

**DOI:** 10.1016/j.clinsp.2023.100226

**Published:** 2023-06-08

**Authors:** Shankargouda Patil, Marcos Roberto Tovani-Palone

**Affiliations:** a College of Dental Medicine, Roseman University of Health Sciences, South Jordan, Utah 84095, USA; b Department of Research Analytics, Saveetha Dental College and Hospitals, Saveetha Institute of Medical and Technical Sciences (SIMATS), Chennai, Tamil Nadu 600077, India

Human ingenuity has drawn Artificial Intelligence (AI) from the realms of science fiction to reality.^1^ Three of the biggest tech conglomerates in the world have recently announced the release of AI models that may reinvent how academic researchers conduct their searches and literature reviews.^2^ With Microsoft integrating AI into its search engine, the once futuristic idea of intelligent search has become a tangible reality. These cutting-edge AI systems, coupled with massive search engines, have harnessed the vast ocean of information and organized it in ways that were once thought impossible. Researchers are no longer forced to navigate the treacherous waters of irrelevant information with no compass to guide them. Scholarly search has been transformed into a voyage of discovery, with the AI system adapting to the researcher's needs in real time.

Google Scholar is the most widely used search engine, enabling researchers, academicians, and students to access a vast pool of information related to their area of study. These search engines rely on algorithms to index and rank billions of web pages and provide relevant results to users based on keyword matching and relevance ranking. Traditional literature search often involves typing in a search string and parsing through the results. Researchers have to trek through a wide range of academic sources, including peer-reviewed journals, conference proceedings, and academic publishers, and examine the search results based on their relevance and significance.

AI can be used to perform such cumbersome, repetitive tasks, freeing human capital to work on higher-impact problems. It can process more information more quickly than a human, finding patterns and discovering relationships in data that a human researcher may miss.^3^ By leveraging their ability to process and analyze large amounts of text-based data, Large Language Models (LLMs) have the potential to serve as an effective tool for researchers.^4^ LLMs correspond to AI systems that have been trained to generate and manipulate text. LLMs are trained on massive amounts of data, typically drawn from the internet, books, and other written materials to learn patterns and relationships within the data. These models, based on deep neural networks, are capable of producing coherent and semantically meaningful text that is indistinguishable from text written by humans. They can assist in retrieving relevant literature by utilizing their vast understanding of language to provide search results that are more precise and relevant than traditional keyword-based search engines, serving up information in clear simple sentences rather than as a pile of internet links that need to be explored further.^5^

An LLM integrated with a search engine analyses a scholarly literature search query and understands the context of the search, such as the author, publication year, and research area, allowing it to provide more accurate and relevant results, cutting down the time spent on manual literature searches. They can provide explanations and additional information in response to follow-up questions, allowing researchers to quickly locate relevant information.^6^ This level of interactivity is not possible with traditional search engines.

A researcher exploring an unfamiliar topic can use a language model to process a large corpus of papers and extract the keywords and named entities, and then use these terms as the basis for a query. The algorithms can analyze the citations in various papers and identify emerging trends, highlighting papers that are particularly relevant. This can significantly speed up the literature review process, generating pertinent search results that match the researcher's specific needs, and saving time and effort.

LLMs can also be used to perform summarization tasks, which can assist researchers in quickly reviewing the contents of large numbers of papers and rapidly assessing their relevance to their research inquiry. In this context, AI systems are able to generate summaries and extract and categorize relevant information (findings, numerical data, text data, image data) from papers, which can provide researchers with a concise overview of their contents. The extracted data can be used to generate insights and inform analysis. Such apps powered by AI, such as Paper Digest and Penelope.ai, are already helping academics get their research published faster.

However, it is important to note that AI language models are not perfect and may make errors or inaccuracies in their predictions. These factual errors can have far-reaching consequences, as Google's parent company Alphabet found to its dismay. Its flagship AI-language model, titled 'Bard,' made an elementary factual error in its demonstration video, causing the company's shares to drop approximately 100 billion dollars in market value. These errors are largely due to faulty training data used to train the model. If the training data contains inaccuracies or biases, these may be incorporated into the model's predictions, leading to errors. For example, if a language model is trained on news articles that contain inaccuracies, it may report those inaccuracies in its predictions.

Moreover, the sheer size and complexity of language models can also contribute to errors. As language models become larger and more complex, it becomes increasingly difficult to evaluate and validate their predictions thoroughly and ensure that they make accurate and reliable inferences. A researcher using AI-powered search engines must exercise due diligence by thoroughly verifying the veracity and accuracy of all obtained data prior to utilization.

In short, the power of LLMs has been rising and their impact on academic research is set to become more profound. By leveraging the power of AI, search engines can now provide results that go beyond simple keyword matches and instead take into account the meaning and context of the search query. This combination can help to simplify many of the complex tasks involved in scholarly research, allowing researchers to focus on the core objectives of their work and achieve their research goals more effectively. Despite this, caution is needed. If on the one hand, the possibilities are almost endless, on the other, all inherent limitations must also be taken into account. In light of this, human innovation and critical thinking should not be replaced as a whole, given that many of the inconsistencies and perceptions of the real world are not plausible to be managed by machines or new technologies, such as AI.^7^^,^^8^

## Declaration of Competing Interest

The authors declare no conflicts of interest.
